# Reducing the use of inhaled corticosteroids in mild-moderate COPD: an observational study in east London

**DOI:** 10.1038/s41533-020-00191-y

**Published:** 2020-07-31

**Authors:** James N. Cole, Rohini A. Mathur, Sally A. Hull

**Affiliations:** Centre for Primary Care and Public Health, Queen Mary University of London, London, UK

**Keywords:** Therapeutics, Respiratory tract diseases

## Abstract

Inhaled corticosteroids (ICS) are often prescribed for worsening breathlessness, exacerbation frequency or lung function in chronic obstructive pulmonary disease (COPD). In mild-moderate disease and infrequent exacerbations, treatment risks may outweigh benefits and ICS may be withdrawn safely under supervision. A systematic ICS deprescribing programme for patients with mild-moderate COPD was introduced in an east London Clinical Commissioning Group (CCG) in April 2017. Primary care patient record analysis found that prescribing fell from 34.9% (*n* = 701) in the 18 months pre-intervention to 26.9% (*n* = 538) by the second year of implementation, decreasing 0.84% per quarter post intervention (*p* = 0.006, linear regression). The relative decrease was greater than the comparison CCG (23.0% vs. 9.9%). Only South Asian ethnicity was associated with increased cessation (odds ratio 1.48, confidence interval (CI) 1.09–2.01), *p* = 0.013, logistic regression). Patient outcome data were not collected. A primary care-led programme comprising local education, financial incentivisation and consultant support led to a significant decrease in ICS prescribing.

## Introduction

Inhaled corticosteroids (ICS) have been used extensively in the management of chronic obstructive pulmonary disease (COPD) over the past 40 years, derived originally from their use in asthma. The use of ICS in milder COPD has become increasingly controversial. Randomised controlled trials (RCTs) have demonstrated that ICS can reduce the frequency of exacerbation, preserve lung function and slow the decline in quality of life, but are also associated with higher prevalence of pneumonia, oral candidiasis and voice hoarseness^1^. Other risks such as reduced bone density, cataract and adrenal suppression have also been postulated^2,3^.

The benefits of ICS are greatest in severe disease, where forced expiratory volume in one second (FEV1) is <50% predicted, or exacerbations are frequent (two or more per year). Others who are likely to benefit include patients with a coexisting diagnosis of asthma (asthma–COPD overlap syndrome), a history of atopy or high blood eosinophils^4^. Those patients with mild-to-moderate airflow obstruction on spirometry and infrequent exacerbations may experience greater risks than benefits.

For over a decade, international COPD guidelines from the Global Initiative for Chronic Obstructive Lung Disease (GOLD) have advocated a risk stratification approach to inform ICS therapy (https://goldcopd.org/gold-reports). Although subsequent versions have moved away from using spirometry to guide pharmacological treatment, guidance at the inception of the project (2016) reserved ICS for more severe cases based on spirometry and exacerbation rate. GOLD advised that management of mild-moderate cases (predicted FEV1 > 50%, with fewer than two COPD exacerbations and no COPD-related hospital admissions in the preceding year) should focus on adequate bronchodilator therapy with long-acting *β-*agonist (LABA) and long-acting muscarinic antagonist (LAMA) inhalers, alongside influenza vaccination, smoking cessation and pulmonary rehabilitation. In contrast, UK guidance at the time from the National Institute for Health and Care Excellence (NICE, 2010 guidance) promoted the use of ICS based on symptomatology regardless of spirometry or exacerbation rate (https://www.nice.org.uk/guidance). Since its updated guidance in 2018, NICE now primarily reserves ICS for patients who have features of asthma, frequent exacerbations or features suggesting steroid responsiveness. However, it does promote ICS for patients who remain symptomatic despite optimal bronchodilator therapy.

ICS continue to be prescribed in mild-to-moderate COPD despite the adverse risk–benefit profile; however, prescribing rates have started to decline over the past 10 years^5,6^.

Deprescribing of inappropriate ICS has attracted increasing attention over recent years. A growing number of RCTs^7–12^ and real-world studies^13,14^ have explored the effects of ICS withdrawal on breathlessness, quality of life, medication use, lung function and exacerbation rates. Most of these have studied heterogeneous patient groups across the spectrum of COPD severity. Systematic reviews have demonstrated small deteriorations in quality of life, lung function and exacerbation frequency, but not to a statistically or clinically significant degree^15–17^. Studies examining ICS withdrawal in the mild-moderate group (predicted FEV > 50%) showed no deterioration in these variables provided bronchodilator therapy was maintained^12,13^.

At the start of this project, there was no internationally agreed process for ICS withdrawal, although approaches using risk stratification based on predicted FEV1 and infrequent exacerbation rates have been suggested^18,19^. ICS withdrawal has been shown to be acceptable to patients in this low-risk group given adequate explanation^20^.

Systematic programmes to achieve ICS reduction have been undertaken in other parts of the United Kingdom, overseen by respiratory consultants running virtual clinics in general practice^21^. The aim of this study is to evaluate a quality improvement programme to reduce ICS prescribing in primary care in east London. Specific objectives include:

- Comparing aggregate practice rates of ICS prescribing pre- and post intervention among individuals with mild-moderate COPD.
- Using an embedded case–control study to identify individual level predictors of ICS cessation within the mild-moderate COPD population.
- Estimating the prescribing cost savings associated with the programme.

## Results

At the start of the pre-intervention period in December 2015, there were 3918 patients on COPD GP practice registers in Tower Hamlets from a total registered population of 309,054 (1.3%), and 3392 patients on COPD registers in City and Hackney from a registered population of 402,087 (0.8%). Excluding those with a historical asthma diagnosis and all those with a predicted FEV1 of 50% or below on their most recent spirometry left a study population of 2009 individuals in Tower Hamlets and 1487 individuals in City and Hackney (see flow chart: Supplementary Fig. 1).

Within the mild-moderate COPD intervention cohort, at baseline 87.0% (*n* = 1748) had a FEV1/forced vital capacity (FVC) ratio < 0.7; 11.9% (*n* = 240) had a FEV1/FVC ratio ≥ 0.7; 1.0% (*n* = 21) had no FEV1/FVC recorded in the GP record.

In the first pre-intervention quarter, among those people with mild-moderate COPD, 701 (34.9%) individuals in Tower Hamlets and 493 (33.2%) in City and Hackney were prescribed ICS.

**Fig. 1:**
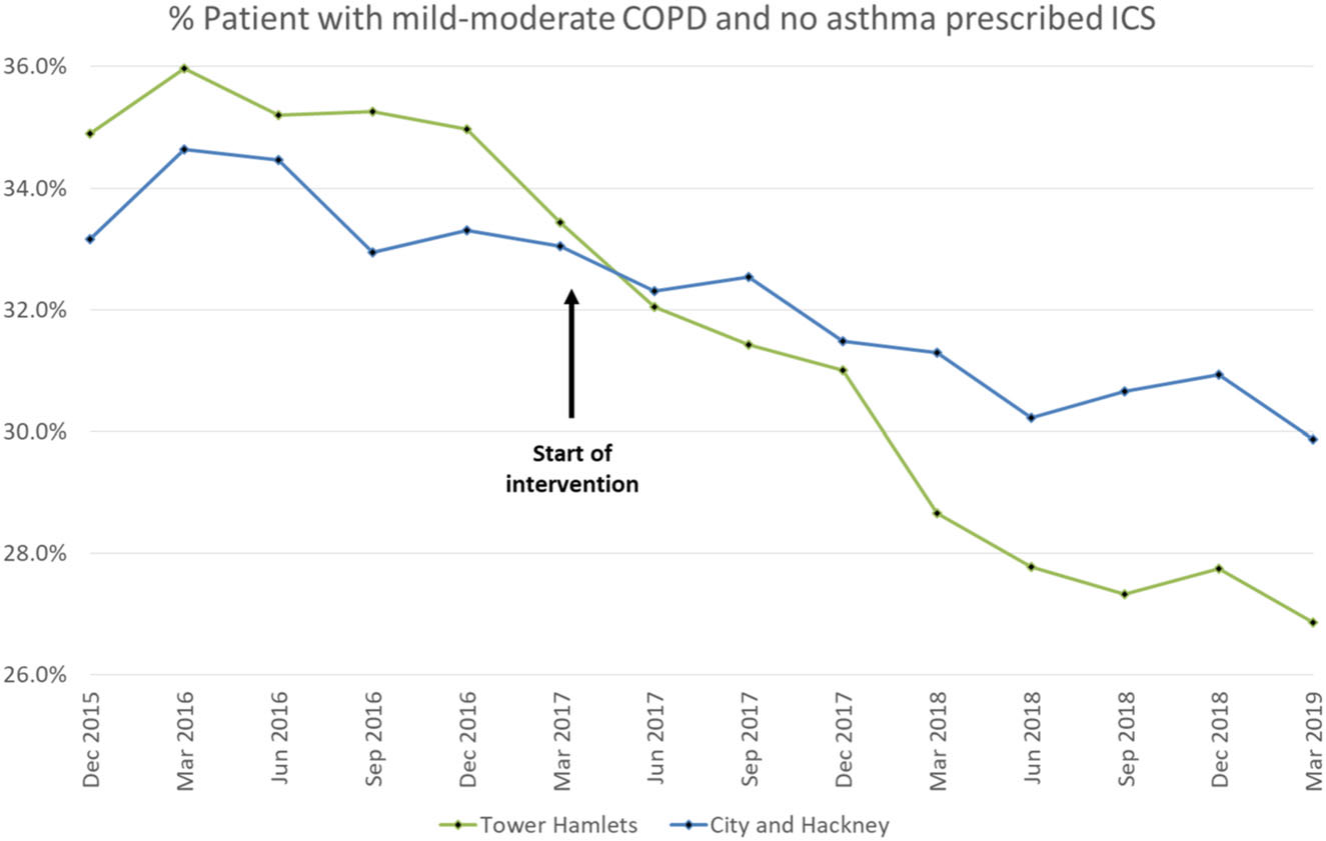
% Patient with mild-moderate COPD and no asthma prescribed ICS. ICS prescribing rates in Tower Hamlets (intervention CCG) and City and Hackney (comparison CCG).

By the final quarter of the 2-year post-intervention period, prescribing rates in the mild-moderate COPD group fell to 26.9% (*n* = 538/2003) in Tower Hamlets—a relative decrease of 23.0%. The proportion of patients with mild-moderate COPD prescribed ICS in City and Hackney fell to 29.9% (*n* = 489/1637) in the final post-intervention quarter—a relative decrease of 9.9% (see Fig. 1).

ICS prescribing in the intervention CCG was stable during the 18-month pre-intervention period, with no significant change in the proportion of people prescribed an ICS between each quarter (*β* −0.06% per quarter, *p* = 0.701, linear regression). The proportion of individuals prescribed an ICS declined significantly by 0.84% per quarter in the final 18 months post intervention (*p* < 0.001, linear regression). Regression analysis comparing the slope of decline before and after the intervention showed that the rate of decline accelerated significantly post intervention (*p* = 0.006, linear regression) (see Table 1).

**Table 1:** ICS prescribing rates in Tower Hamlets (intervention CCG) and City and Hackney (comparison CCG).

	Quarterly change in ICS prescription	*P* value for quarterly change	*P* value for difference in slopes pre- and post intervention
Pre-intervention (Dec 2015–Mar 2017)	−0.06%	0.789	0.006
Post intervention (Apr 2017–Mar 2019)	−0.84%	<0.001	
	**% ICS in TH post intervention**	**% ICS in CH post intervention**	**Difference between CCGs, %*p*value**
	29.1	31.2	2.1, 0.022

Prescribing rates in the intervention CCG (Tower Hamlets) were significantly lower than in the comparison CCG (City and Hackney) in the post-intervention period. Comparing prescribing in the final 18 months post intervention showed the average proportion of individuals prescribed an ICS was 2.5% lower in the intervention CCG (*p* < 0.003, linear regression).

Patients were classified as having stopped ICS if they were prescribed an inhaler in one-quarter, but then not prescribed in the three subsequent quarters. Using this definition, 23.6% (*n* = 219) of those with mild-moderate COPD in Tower Hamlets stopped ICS, while 76.4% (*n* = 708) continued (see Table 2). Using multivariable logistic regression adjusted for all predictors of interest, deprivation score, and clustering by practice, we found that South Asian ethnicity was associated increased odds of ICS cessation (odds ratio (OR) South Asian vs. white 1.48, 95% confidence interval (CI) 1.08–2.01, *p* = 0.013, logistic regression), while having two or more comorbidities was associated with decreased odds of with ICS cessation (OR 2+ comorbidities vs. none 0.27, 95% CI 0.14–0.51, *p* < 0.001, logistic regression). Age, sex and baseline smoking status were not associated with ICS cessation (see Table 3).

**Table 2:** Baseline characteristics of people who continue and stop inhaled corticosteroids.

	Non-stoppers	Stoppers
	*N* (%)	*N* (%)
Denominator	708	219
Age at baseline (SD)	68.2 (11.8)	69.1 (12)
<60 years	169 (23.9)	47 (21.5)
≥60 years	539 (76.1)	172 (78.5)
Gender		
Male	422 (59.6)	141 (64.4)
Female	286 (40.4)	78 (35.6)
Ethnic Group		
White	513 (72.5)	142 (64.8)
South Asian	148 (20.9)	62 (28.3)
Black	18 (2.5)	7 (3.2)
Other/unknown	29 (4.1)	8 (3.7)
Deprivation quintile		
1 (least deprived)	142 (20.1)	44 (20.2)
2	142 (20.1)	44 (20.2)
3	136 (19.2)	47 (21.6)
4	151 (21.4)	41 (18.8)
5 (most deprived)	136 (19.2)	42 (19.3)
Smoking Status		
Current non-smoker	405 (57.7)	140 (64.2)
Current smoker	297 (42.3)	78 (35.8)
Baseline comorbidities		
Atrial fibrillation	50 (7.1)	24 (11)
Cancer	70 (9.9)	22 (10)
CHD	146 (20.6)	54 (24.7)
CKD	131 (18.5)	44 (20.1)
Dementia	16 (2.3)	6 (2.7)
Depression	192 (27.1)	54 (24.7)
Diabetes	182 (25.7)	64 (29.2)
Epilepsy	13 (1.8)	4 (1.8)
Heart failure	42 (5.9)	25 (11.4)
Hypertension	341 (48.2)	106 (48.4)
Osteoporosis	46 (6.5)	12 (5.5)
Peripheral arterial disease	46 (6.5)	6 (2.7)
Rheumatoid arthritis	18 (2.5)	9 (4.1)
Serious mental illness	21 (3)	9 (4.1)
Stroke	48 (6.8)	16 (7.3)

^a^Baseline is date of first ICS prescription during post-intervention period.

**Table 3:** Odds of stopping inhaled corticosteroids.

Predictor	Stoppers	Non-stoppers	OR (95%CI) *p* value
Age			
<60 years (ref.)	169 (23.9)	47 (21.5)	1.00
≥60 years	539 (76.1)	172 (78.5)	1.11 (0.84,1.47) 0.474
Gender			
Male (ref.)	422 (59.6)	141 (64.4)	1.00
Female	286 (40.4)	78 (35.6)	1.12 (0.84,1.51) 0.432
Ethnic group			
White (ref.)	513 (72.5)	142 (64.8)	1.00
South Asian	148 (20.9)	62 (28.3)	1.48 (1.08,2.01) 0.013
Black	18 (2.5)	7 (3.2)	1.42 (0.70,2.85) 0.330
Other/unknown	29 (4.1)	8 (3.7)	1.04 (0.46,2.32) 0.932
Smoking status			
Never/ex-smoker (ref.)	405 (57.7)	140 (64.2)	0.80 (0.56,1.17) 0.252
Current smoker	297 (42.3)	78 (35.8)	0.84 (0.51,1.40) 0.507
Number of QOF comorbidities			
0	491 (69.4)	140 (63.9)	0.92 (0.53,1.58) 0.759
1	180 (25.4)	63 (28.8)	1.01 (0.70,1.46) 0.943
2+	37 (5.2)	16 (7.3)	0.27 (0.14,0.51) <0.001

^a^Model accounts for all factors reported in the table, and additionally, clustering by practice and Townsend deprivation quintile.

EMIS Web provides an estimated National Health Service (NHS) cost for each medication extracted. The estimated annual cost of ICS inhaler products decreased from £640,309 pre-intervention to £532,732 post intervention—a reduction of 17%.

## Discussion

This study demonstrates that a programme providing clinical guidance and education, IT support and financial incentivisation to networks of GP practices is associated with a significant acceleration in the reduction of ICS prescribing for those with mild-moderate COPD.

The programme was innovative in that primary care clinicians, rather than secondary care respiratory specialists, were enabled to lead the withdrawal process. The evaluation demonstrates that such a strategy is possible, provided that adequate community-based clinical support from respiratory specialists is available if required. Such programmes can also lead to substantial cost savings.

The study used retrospective data from electronic health records. The population of east London is comparatively mobile and GP practices can see up to a quarter of their patients change in 1 year. This was reflected in our data where significant numbers of patients were not registered for at least one-quarter of the study period. This may have underestimated the number of COPD patients for each quarter, but it is not evident that they would be more or less likely to be eligible for, or to engage with, an ICS reduction programme.

Tower Hamlets has a history of introducing quality assurance to spirometry performed in practice settings^22^. In the intervention group, 99% of individuals had a recorded FEV1/FVC ratio. However, 12% had a value falling above the obstructive threshold of <0.7. Some of these patients, particularly those <60 years of age, will have COPD with a FEV1/FVC ratio of >0.7, as the lower limit of normal for FEV1/FVC varies with age^23^. There may have been cases where spirometry results from hospital were not transcribed into the GP record. Additionally, there may be some diagnostic error in this routinely derived data set.

The local clinical guidance, which supported this programme, encouraged clinicians to review eosinophil counts and exacerbation rates; however, these criteria were not used to define the cohort in the evaluation. Exacerbations and hospitalisations are variably coded in the primary care record, meaning that including these factors would reduce the reliability of the analysis.

No data were collected on clinical outcomes, which might include adverse effects such as increased exacerbation rate or reduced lung function following ICS reduction. The local clinical guideline recommended regular clinical review during the withdrawal phase, and provided advice in the event of clinical deterioration. Clinicians were advised to restart ICS if appropriate and seek advice from a respiratory physician.

Cost data for ICS products was based on 2019 prices. Reductions in cost may have been due to the promotion of more cost-effective brands of inhaler (particularly ICS/LABA combinations) in addition to the reduction in volume of prescriptions related to the ICS reduction programme.

The declining secular trend in ICS prescribing was reflected in our study. Given the primary care-led approach of this ICS reduction programme, the authors could find no similar studies for comparison of effectiveness. RCT and real-world studies provide data on patient adherence to ICS withdrawal; however, it is not possible to infer from our data whether a patient did not withdraw ICS because of the lack of appropriate clinical review, patient dissent or attempted but abandoned withdrawal.

We found that ICS cessation was more successful in ethnic minority groups, particularly patients of South Asian origin. Black ethnicity was associated with a modest, but not statistically significant increase in ICS cessation, possibly as a result of the small numbers of patients in the study. This may be as a result of health beliefs, which favour non-pharmacological intervention^24^. Deprescribing was less successful in patients with greater comorbidity, which may be attributable to the increasing complexity of clinical encounters and number of healthcare professionals involved^25^.

The development of managed practice networks in Tower Hamlets since 2009 has been a key contextual factor in the delivery and rapid implementation of quality improvement programmes. Practice networks have the potential to improve clinical performance through mechanisms of peer-to-peer support and because financial incentives are directed at network, rather than individual practice, performance^22^. The development of primary care networks is a current NHS priority.

This evaluation demonstrates that a primary care-led programme of ICS reduction is possible, well received by practice clinicians and provides significant cost saving. This programme could be replicated in primary care in other parts of the United Kingdom, with significant potential benefits in terms of preventing adverse effects of ICS therapy and improving the cost-effectiveness of COPD management.

## Methods

### Setting

East London is home to a young, ethnically diverse population characterised by high levels of deprivation and multimorbidity. Smoking prevalence and COPD admission rates are among the highest in London (https://fingertips.phe.org.uk/profile/inhale). In Tower Hamlets, the intervention clinical commissioning group (CCG), each practice is part of a geographic network and receives additional funding through contracts for enhanced services based on chronic disease indicators including COPD.

### Intervention

A quality improvement programme to promote the stepwise withdrawal of ICS was launched in the east London locality of Tower Hamlets in April 2017. The aim of the programme was to provide tools and support to GP practice clinicians to undertake ICS withdrawal in primary care. All 35 practices, with a GP registered population of 347,054 in this CCG took part. A neighbouring CCG, City and Hackney, acted as a natural control. All 76 GP practices in Tower Hamlets and City and Hackney use the EMIS Web clinical system and hold information sharing agreements with the Clinical Effectiveness Group (CEG), based at Queen Mary University of London. CEG extract pseudonymised patient data to promote quality improvement through evidence-based guidelines, standardised data entry templates and clinical dashboards (https://www.qmul.ac.uk/blizard/ceg/).

**Fig. 2:**
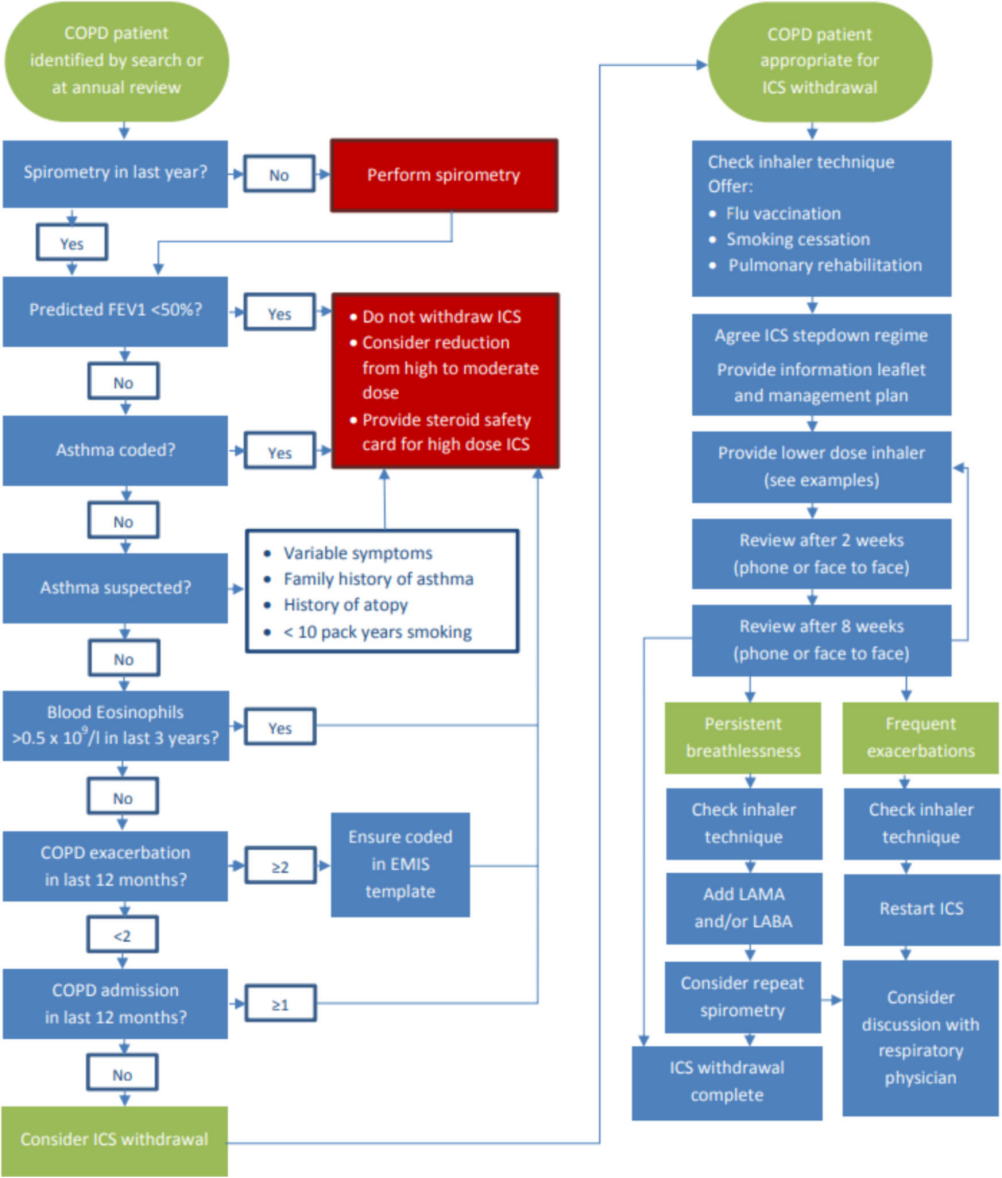
Algorithm to support primary care clinicians make clinical management decisions relating to ICS therapy. Patient identification and management of ICS Withdrawal Algorithm provided to clinicians within local clinical guideline.

The intervention components included:

- A brief clinical guideline developed through the CCG COPD working group in collaboration with local stakeholders from primary care, consultant respiratory physicians from the local hospital NHS trust and the Tower Hamlets Public Health team. The guidance was based on clinical evidence and programmes successfully undertaken elsewhere^21^, and included an algorithm to support clinical decision making (see Fig. 2) based on the risk assessment model from GOLD. Patients with severe or very severe disease on spirometry (predicted FEV1 < 50%), known or suspected asthma, eosinophilia, more than one COPD exacerbation or any COPD admission in the past 12 months were excluded from the intervention. For patients deemed potentially suitable for ICS withdrawal, the algorithm prompted the clinician to consider inhaler technique, influenza vaccination, smoking cessation and pulmonary rehabilitation as part of clinical management. Regular clinical review was encouraged throughout the ICS withdrawal period. The guidance provided examples of stepwise reduction of ICS inhaler potency, along with suggested actions if the patient experienced worsening breathlessness or increasing exacerbation frequency during withdrawal.
  We developed an accompanying patient information leaflet, which was piloted at a pulmonary rehabilitation group and distributed to local practices (see Supplementary Fig. 2). The programme was presented at CCG COPD study days, which were attended by primary care clinicians from across the borough. Quarterly multidisciplinary meetings were organised in each network and were attended by primary care clinicians from each practice and a Consultant Respiratory Physician from the local NHS trust. These meetings provided primary care clinicians with opportunities for education about COPD and the ICS withdrawal programme, as well as a forum to discuss specific clinical cases.
- Practices were provided with two electronic tools within EMIS Web to identify patients potentially eligible for ICS reduction: pop-up consultation alerts to notify the clinician during a clinical contact and searches to generate a list of patients for who COPD review may be prioritised.
- Practices were financially incentivised to achieve a reduction of 20% in ICS prescribing in the target group between April 2017 and March 2018. Incentives continued in the next financial year (2018–19) based on a threshold target of ICS prescribing rather than a reduction. This was to avoid penalising practices, which had already made significant reductions. Practice performance against these indicators compared to all other practices in the CCG was fed back via monthly dashboards.

### Data sources

Evaluation began 2 years into the programme in May 2019. Patient level data were extracted for all individuals in Tower Hamlets and City and Hackney with diagnosed COPD (as defined by the UK Quality and Outcomes Framework (QOF) (https://digital.nhs.uk/data-and-information/data-collections-and-data-sets/data-collections/quality-and-outcomes-framework-qof) on a quarterly basis between December 2015 and April 2019. Individuals with a prior diagnosis of asthma were not included in the study population.

Coded practice data relating to patient demographics, disease characteristics (smoking status, diagnostic FEV1/FVC ratio, and latest predicted FEV1%), QOF comorbidities (Atrial Fibrillation, Cancer, Coronary Heart Disease, Chronic Kidney Disease, Dementia, Depression, Diabetes, Epilepsy, Heart Failure, Hypertension, Osteoporosis, Peripheral Arterial Disease, Rheumatoid Arthritis, Serious Mental Illness and Stroke) and ICS prescribing were extracted for the six quarters before and eight quarters after the intervention (see Supplementary Table 1). All data were de-identified and managed according to the UK NHS information governance requirements. Ethical approval was not required for this report as it relies on the use of routinely recorded de-identified data published in aggregate form. All GPs in the participating east London practices consented to the use of their anonymised patient data for research and development for patient benefit.

### Statistical analysis

An interrupted time-series analysis examined the proportion of individuals with mild-moderate COPD (defined as their latest FEV1 > 50%) prescribed an ICS in each quarter of the study period. Linear regression was employed to compare the quarterly change in prescribing for the pre-intervention period (Q3 financial year 2015–2016—Q4 financial year 2016–2017) to that in the post-intervention period (Q1 financial year 2017–2018—Q4 financial year 2018–2019).

Within the intervention CCG of Tower Hamlets, a nested case–control study was conducted to identify predictors of ICS cessation. Individuals with mild-moderate COPD prescribed an ICS at any point during follow-up were eligible for inclusion. Cases were defined as individuals who had ceased ICS for at least 9 months (three quarters) following a first ICS prescription in the study period. Controls were defined as individuals who were prescribed an additional ICS at any point over the 9 months following their index prescription. Demographic and comorbidity data were collected at the time of first ICS prescription. Individuals with <9 months of follow-up were excluded from the study; reasons for this included de-registering from their GP practice during the study period, or having their first ICS prescription <9 months before the end of the follow-up period.

Multivariable logistic regression analysis was used to identify predictors of ICS cessation. Predictors of interest included age, gender, ethnicity, smoking status, comorbidity (measured as count of QOF conditions at the time of first ICS prescription). The models additionally adjusted for Townsend deprivation quintile and clustering by practice. All analyses were completed using Stata version 15.

### Reporting summary

Further information on research design is available in the Nature Research Reporting Summary linked to this article.

## Supplementary information

Supplementary Information

Reporting Summary

## Data Availability

The datasets generated and analysed during the current study are available from the corresponding author on reasonable request.
