# Supplementary material for: Reducing the use of inhaled corticosteroids in mild-moderate COPD: an observational study in east London
Source: NPJ Prim Care Respir Med. 2020 Jul 31;30:34. doi: 10.1038/s41533-020-00191-y (PMC7395712; doi:10.1038/s41533-020-00191-y)
Supplement: Supplementary file 1 — Supplementary Information [file 41533_2020_191_MOESM1_ESM.pdf]

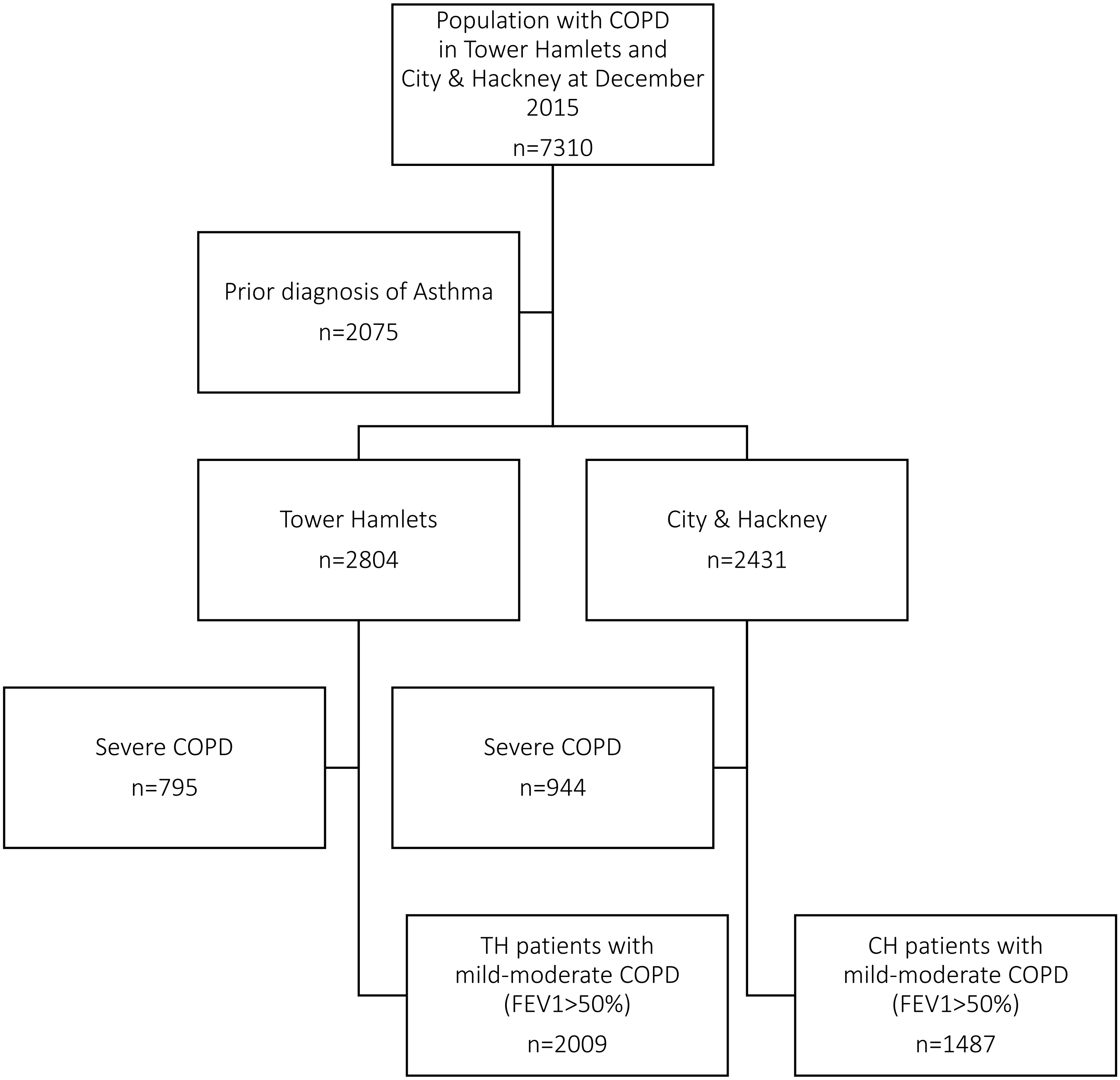

Supplementary Figure 1: Study population flowchart for patients within intervention and comparison CCGs

NHS

Patient Information Leaflet

Reducing and Stopping Inhaled Corticosteroids in COPD

What are inhaled corticosteroids?

Corticosteroid or 'steroid' inhalers are usually prescribed for people with more severe chronic obstructive pulmonary disease (COPD). Examples include fluticasone (which is the steroid present in Seretide®) and budesonide (which is the steroid present in Symbicort®).

Why am I taking them?

Steroids in COPD help prevent acute exacerbations – becoming unwell with worsening cough, phlegm and breathlessness. But recent research suggests steroids may do more harm than good in mild COPD.

What are the risks of continuing steroids?

Using steroid inhalers long term can increase the chance of getting pneumonia. Less serious problems include thrush in the mouth and getting a hoarse voice. Steroids may also increase the chance of osteoporosis (weak bones), cataracts in the eyes, and diabetes.

What are the risks of stopping steroids?

Recent research shows that if you have mild COPD, you are not having frequent exacerbations and you do not have asthma, then it is safe to gradually cut down and then stop your steroid inhaler.

How will this happen?

Your doctor or nurse will advise on changes to your inhaler to gradually reduce the amount of steroid you take. It is important to stay on your other inhalers as these help keep your airways open. Regular reviews at the surgery, or by phone, will help you manage these changes.

What else can I do to help my COPD?

There are other things you can do to manage your COPD and stay healthy:

Flu vaccination | **Stop Smoking** | **Pulmonary Rehabilitation**

Ask your GP or nurse for more information.

Supplementary Figure 2: Patient information leaflet provided as part of ICS reduction programme

Predicted FEV1	339S.
Tobacco consumption	137%
Asthma	H33..% (excluding H333.) , H3120 , H3B.. , 173A.
Atrial Fibrillation	G573%
Cancer	B0...-B32z. , B34..-B6z0. (excluding B677.) , Byu..-Byu41 , Byu5.-ByuE0 , K1323 , K01w1 , 68W24 , C184.
CHD	G3...-G309. , G30B.-G330z (excluding G310.) , G33z.-G3401 , G342.-G35X. , G38..-G3z.. , Gyu3.% (excluding Gyu31)
CKD	1Z12. , 1Z13. , 1Z14. , 1Z15. , 1Z16. , 1Z1B.-1Z1L. , K053. , K054. , K055. , 1Z1T. , 1Z1V. , 1Z1W. , 1Z1X. , 1Z1Y. , 1Z1Z. , 1Z1a. , 1Z1b. , 1Z1c. , 1Z1d. , 1Z1e. , 1Z1f., 1Z10. , 1Z11. , 1Z17.-1Z1A. , K051. , K052. , 1Z1M. , 1Z1Q. , 1Z1N. , 1Z1P. , 1Z1R. , 1Z1S.
COPD	H3... , H31..% (excluding H3101 , H31y0 , H3122) , H32..% , H36..-H3z.. (excluding H3y0. , H3y1.) , H5832 , H4640 , H4641 , Hyu30 , Hyu31
Dementia	Eu02.% , E00..% , Eu01.% , E02y1 , E012.% , Eu00.% , E041. , Eu041 , F110.-F112. , F116. , F118. , F21y2 , A410. , A411.% , Eu107 , F11x7
Depression	E0013 , E0021 , E112.% , E113.% , E118. , E11y2 , E11z2 , E130. , E135. , E2003 , E291. , E2B.. , E2B1. , Eu204 , Eu251 , Eu32.% (excluding Eu32A , Eu32B , Eu329) , Eu33.% , Eu341 , Eu412
Diabetes	C10.. , C109J , C109K , C10C. , C10D. , C10E.% , C10F.% (excluding C10F8) , C10G.% , C10H.% , C10M.% , C10N.% , PKyP. , C10P.% , C10Q.
Epilepsy	F25..% (excluding F2501 , F2504 , F2511 , F2516 , F256.% , F258.-F25A. , F25y4 , F25G. , F25H.) , F1321 , SC200
Heart Failure	G58..% , G1yz1 , 662f.-662i.
Hypertension	G2... , G20..% , G24..-G2z.. (excluding G24z1 , G2400 , G2410 , G27..) , Gyu2. , Gyu20
Osteoporosis	N330.% (excluding N3308 , N3309) , N3312 , N3313 , N3316 , N3318–N331B , N331H–N331M , NyuB0 , NyuB1 , NyuB8 , N3314 , N3315 , N3746 , NyuB2
Palliative Care	1Z01. , 2JE.. , 2Jf.. , 38VY. , 38Vb. , 38Vd. , 38Ve. , 38Vf. , 38Vg. , 38Vh. , 38Vi. , 8BA2. , 8BAP. , 8BAS. , 8BAT. , 8BAe. , 8BJ1. , 8CM1.% (excluding 8CM15) , 8CM4. , 8CME. , 8CMj. , 8CMk. , 8H6A. , 8H7L. , 8H7g. , 8HH7. , 8IEE. , 9EB5. , 9Ng7. , ZV57C , 8CMQ. , 9NgD. , 9G8.. , 9c0P. , 9c0N. , 8CMW3 , 9K9.. , 9367. , 9c0L0 , 9c0M. , 9NNd. , 8CMb. , 8B2a. , 9NNf0 , 38QH. , 38QK. , 8CMg. , 2Jg.., 9NNq. , 9NNr. , 9NNs.
Peripheral Arterial Disease	G73.. , G73z.% (excluding G73z1) , Gyu74 , G734. , G73y.
Rheumatoid Arthritis	N040.% , N041. , N042.% (excluding N0420) , N047. , N04X. , N04y0 , N04y2 , Nyu11 , Nyu12 , Nyu1G , Nyu10 , G5yA. , G5y8.
Serious Mental lness	E10..% , E110.% , E111.% , E1124 , E1134 , E114.-E117z , E11y.% (excluding E11y2) , E11z. , E11z0 , E11zz , E12..% , E13..% (excluding E135.) , E2122 , Eu2..% , Eu30.% , Eu31.% , Eu323 , Eu328 , Eu333 , Eu32A , Eu329
Stroke	G61..% (excluding G617.) , G63y0-G63y1 , G64..% , G66..% (excluding G669.) , G6760 , G6W.. , G6X.. , Gyu62-Gyu66 , Gyu6F , Gyu6G, G65..-G654. , G656.-G65zz , ZV12D , Fyu55

Supplementary Table 1: Read codes extracted from primary care patient records
