# Supplementary material for: Reducing the use of inhaled corticosteroids in mild-moderate COPD: an observational study in east London
Source: NPJ Prim Care Respir Med. 2020 Jul 31;30:34. doi: 10.1038/s41533-020-00191-y (PMC7395712; doi:10.1038/s41533-020-00191-y)
Supplement: Supplementary file 2 — Reporting Summary [file 41533_2020_191_MOESM2_ESM.pdf]

nature research

Corresponding author(s): James Cole

Last updated by author(s): Mar 5, 2020

Reporting Summary

Nature Research wishes to improve the reproducibility of the work that we publish. This form provides structure for consistency and transparency in reporting. For further information on Nature Research policies, seeAuthors & Referees and theEditorial Policy Checklist .

Statistics

For all statistical analyses, confirm that the following items are present in the figure legend, table legend, main text, or Methods section.

n/a	Confirmed	
☐	☒	The exact sample size (*n*) for each experimental group/condition, given as a discrete number and unit of measurement
☐	☒	A statement on whether measurements were taken from distinct samples or whether the same sample was measured repeatedly
☐	☒	The statistical test(s) used AND whether they are one- or two-sided<br>*Only common tests should be described solely by name; describe more complex techniques in the Methods section.*
☐	☒	A description of all covariates tested
☐	☒	A description of any assumptions or corrections, such as tests of normality and adjustment for multiple comparisons
☐	☒	A full description of the statistical parameters including central tendency (e.g. means) or other basic estimates (e.g. regression coefficient) AND variation (e.g. standard deviation) or associated estimates of uncertainty (e.g. confidence intervals)
☐	☒	For null hypothesis testing, the test statistic (e.g. *F*, *t*, *r*) with confidence intervals, effect sizes, degrees of freedom and *P* value noted<br>*Give P values as exact values whenever suitable.*
☒	☐	For Bayesian analysis, information on the choice of priors and Markov chain Monte Carlo settings
☒	☐	For hierarchical and complex designs, identification of the appropriate level for tests and full reporting of outcomes
☒	☐	Estimates of effect sizes (e.g. Cohen's *d*, Pearson's *r*), indicating how they were calculated

Our web collection on statistics for biologists contains articles on many of the points above.

Software and code

Policy information about availability of computer code

Data collection	EMIS Web (EMIS Health)
Data analysis	Stata version 15 (StataCorp)

For manuscripts utilizing custom algorithms or software that are central to the research but not yet described in published literature, software must be made available to editors/reviewers. We strongly encourage code deposition in a community repository (e.g. GitHub). See the Nature Research guidelines for submitting code & software for further information.

Data

Policy information about availability of data

All manuscripts must include a data availability statement. This statement should provide the following information, where applicable:

- Accession codes, unique identifiers, or web links for publicly available datasets
- A list of figures that have associated raw data
- A description of any restrictions on data availability

The datasets generated and analysed during the current study are available from the corresponding author on reasonable request

Field-specific reporting

Please select the one below that is the best fit for your research. If you are not sure, read the appropriate sections before making your selection.

☒ Life sciences ☐ Behavioural & social sciences ☐ Ecological, evolutionary & environmental sciences

For a reference copy of the document with all sections, see nature.com/documents/nr-reporting-summary-flat.pdf

Life sciences study design

All studies must disclose on these points even when the disclosure is negative.

Sample size	Sample size in this study was based on the exisiting population in two Clinical Commissioning Group (CCG) areas in east London. At the start of the pre-intervention period in December 2015, there were 3,918 patients on COPD GP practice registers in Tower Hamlets (intervention CCG) from a total registered population of 309,054 (1.3%), and 3,392 patients on COPD registers in City & Hackney (comparison CCG) from a registered population of 402,087 (0.8%). Excluding those with a historical asthma diagnosis and all those with a predicted FEV1 of 50% or below on their most recent spirometry, left a study population of 2,009 individuals in Tower Hamlets and 1,487 individuals in City & Hackney (see flow chart: supplementary figure 1).
Data exclusions	Patients with a historical asthma diagnosis and all those with a predicted FEV1 of 50% or below on their most recent spirometry were excluded as the study examined patients with mild-moderate COPD.
Replication	Not applicable.
Randomization	Not applicable. Subjects were not randomized, intervention was not controlled.
Blinding	Blinding not applicable.

Reporting for specific materials, systems and methods

We require information from authors about some types of materials, experimental systems and methods used in many studies. Here, indicate whether each material, system or method listed is relevant to your study. If you are not sure if a list item applies to your research, read the appropriate section before selecting a response.

Materials & experimental systems

n/a	Involved in the study
☒	☐ Antibodies
☒	☐ Eukaryotic cell lines
☒	☐ Palaeontology
☒	☐ Animals and other organisms
☒	☐ Human research participants
☐	☒ Clinical data

Methods

n/a	Involved in the study
☒	☐ ChIP-seq
☒	☐ Flow cytometry
☒	☐ MRI-based neuroimaging

Clinical data

Policy information about clinical studies

All manuscripts should comply with the ICMJEguidelines for publication of clinical research and a completedCONSORT checklist must be included with all submissions.

Clinical trial registration	Study not registered. It is a service improvement project analysing routinely collected anonymised data extracted using existing information sharing agreements between Queen Mary University of London and local GP practices.
Study protocol	Not applicable. It is a service improvement project analysing routinely collected data extracted using existing information sharing agreements between Queen Mary University of London and local GP practices.
Data collection	Patient level data was extracted for all individuals in Tower Hamlets and City & Hackney with diagnosed COPD (as defined by the UK Quality and Outcomes Framework (QOF) on a quarterly basis between December 2015 and April 2019. Individuals with a prior diagnosis of asthma were not included in the study population.
Outcomes	Coded practice data relating to patient demographics, disease characteristics (smoking status, diagnostic FEV1/FVC ratio, and latest predicted FEV1%), QOF co-morbidities (Atrial Fibrillation, Cancer, Coronary Heart Disease, Chronic Kidney Disease, Dementia, Depression, Diabetes, Epilepsy, Heart Failure, Hypertension, Osteoporosis, Peripheral Arterial Disease, Rheumatoid Arthritis, Serious Mental Illness and Stroke) and ICS prescribing were extracted for the 6 quarters before and 8 quarters after the intervention.
